# Supplementary material for: Analysis of PPARγ Signaling Activity in Psoriasis
Source: Int J Mol Sci. 2021 Aug 10;22(16):8603. doi: 10.3390/ijms22168603 (PMC8395241; doi:10.3390/ijms22168603)
Supplement: Supplementary file 1 [file ijms-22-08603-s001.zip › Supplemental materials_Analysis of PPARg signaling activity in psoriasis/Pathway models/Models images and html files/Differentiation of psoriatic T cells/1012573465.html]

PTPN6 ---> TYK2


# ProtModification PTPN6 ---> TYK2

|  |  |
| --- | --- |
| URN | urn:agi-protmodification:in-out:urn:agi-llid:5777:out:urn:agi-llid:7297 |
| Connectivity | 2 |
| References | 12 |
| Effect | unknown |

---
